# Sustained Release of Tacrolimus From a Topical Drug Delivery System Promotes Corneal Reinnervation

**DOI:** 10.1167/tvst.11.8.20

**Published:** 2022-08-19

**Authors:** Simeon C. Daeschler, Kaveh Mirmoeini, Tessa Gordon, Katelyn Chan, Jennifer Zhang, Asim Ali, Konstantin Feinberg, Gregory H. Borschel

**Affiliations:** 1 Neurosciences & Mental Health Program, SickKids Research Institute, Toronto, Ontario, Canada; 2 Division of Plastic and Reconstructive Surgery, The Hospital for Sick Children, University of Toronto, Toronto, Ontario, Canada; 3 Institute of Biomedical Engineering, University of Toronto, Toronto, Ontario, Canada; 4 Department of Ophthalmology and Vision Science, The Hospital for Sick Children, University of Toronto, Toronto, Ontario, Canada; 5 Department of Surgery, Indiana University School of Medicine, Indianapolis, IN, USA; 6 Department of Ophthalmology, Indiana University School of Medicine, Indianapolis, IN, USA

**Keywords:** cornea, corneal innervation, neurotrophic keratopathy, topical therapeutics, drug delivery, tacrolimus, FK506, dry eye, neurotrophic keratitits, corneal ulcer

## Abstract

**Purpose:**

Corneal nerve fibers provide sensation and maintain the epithelial renewal process. Insufficient corneal innervation can cause neurotrophic keratopathy. Here, topically delivered tacrolimus is evaluated for its therapeutic potential to promote corneal reinnervation in rats.

**Methods:**

A compartmentalized neuronal cell culture was used to determine the effect of locally delivered tacrolimus on sensory axon regeneration in vitro. The regenerating axons but not the cell bodies were exposed to tacrolimus (50 ng/mL), nerve growth factor (50 ng/mL), or a vehicle control. Axon area and length were measured after 48 hours. Then, a biodegradable nanofiber drug delivery system was fabricated via electrospinning of a tacrolimus-loaded polycarbonate–urethane polymer. Biocompatibility, degradation, drug biodistribution, and therapeutic effectiveness were tested in a rat model of neurotrophic keratopathy induced by stereotactic trigeminal nerve ablation.

**Results:**

Sensory neurons whose axons were exposed to tacrolimus regenerated significantly more and longer axons compared to vehicle-treated cultures. Trigeminal nerve ablation in rats reliably induced corneal denervation. Four weeks after denervation, rats that had received tacrolimus topically showed similar limbal innervation but a significantly higher nerve fiber density in the center of the cornea compared to the non-treated control. Topically applied tacrolimus was detectable in the ipsilateral vitreal body, the plasma, and the ipsilateral trigeminal ganglion but not in their contralateral counterparts and vital organs after 4 weeks of topical release.

**Conclusions:**

Locally delivered tacrolimus promotes axonal regeneration in vitro and corneal reinnervation in vivo with minimal systemic drug exposure.

**Translational Relevance:**

Topically applied tacrolimus may provide a readily translatable approach to promote corneal reinnervation.

## Introduction

Although corneal innervation is indispensable for ocular protection and surface health, non-invasive therapies that promote corneal reinnervation following corneal nerve fiber loss are currently unavailable. A dense network of corneal nerve fibers provides surface sensation, mediates the blinking reflex, and governs adequate lacrimation to protect the susceptible cornea from injury and desiccation.^1^^,^^2^ To maintain its transparency, the corneal epithelium undergoes a continuous, high-turnover renewal process by replenishing desquamated apical cells with proliferating limbal stem cells.^3^^–^^7^ In response to epithelial injury, these mechanisms of corneal repair, which include stem cell proliferation and centripetal epithelial cell migration, are normally upregulated to rapidly restore ocular surface integrity.^8^ In patients with insufficient corneal innervation, however, these mechanisms are impaired.^9^^,^^10^ As a result, the denervated cornea loses its ability to maintain its surface integrity and to heal epithelial injuries, leading to progressive corneal breakdown and vision loss, termed neurotrophic keratopathy (NK).^9^^,^^11^^–^^13^

The etiology of NK is wide ranging. Surgical or non-surgical trigeminal nerve trauma due to tumor resection, skull fractures, and herpetic infections, as well as congenital disorders (e.g., trigeminal hypoplasia) and systemic diseases (e.g., diabetes), may compromise corneal innervation.^9^^,^^14^ As reliable epidemiological data are scarce,^9^ NK is presently classified as an orphan disease with an estimated prevalence of 1 to 5 individuals in 10,000 (ORPHA: 137596).^15^ However, given the diverse etiology, the actual prevalence may be higher.^16^

The conventional ophthalmic management of NK aims at mitigating symptoms and preventing disease progression but fails to address the fundamental lack of corneal innervation. More recently introduced treatment strategies include topical nerve growth factor (NGF)^17^^–^^20^ application or nerve transfer surgery.^21^^–^^24^ Topical application of recombinant human NGF aims to substitute a hypothesized shortage of NGF in the ocular surface and thereby promote limbal stem cell proliferation and epithelial repair.^18^^,^^25^^,^^26^ Although the corneal surface integrity can be improved in approximately 75% of the patients,^18^^,^^27^ corneal innervation and thereby sensation are not restored.^18^^,^^27^ Nerve transfer surgery, or “corneal neurotization,”^24^ reroutes nearby sensory nerves to the cornea to induce corneal reinnervation by regenerating donor nerve fibers.^13^^,^^21^^,^^28^ Corneal neurotization reliably restores corneal sensation in most patients,^21^^,^^24^ but it is an invasive procedure performed by a specialist surgeon and to which many patients worldwide lack access currently. Therefore, less invasive, and universally available treatment strategies that promote corneal reinnervation could represent substantial progress toward improving the clinical management of patients suffering from corneal nerve fiber loss and NK.

Tacrolimus is a readily available immunosuppressant approved by the U.S. Food and Drug Administration that is used clinically for allergic keratoconjunctivitis,^29^ corneal transplantation,^30^ and other ophthalmic conditions.^31^ Apart from its immunosuppressive effect, tacrolimus promotes axon regrowth in vitro^32^^–^^35^ and axonal regeneration following nerve injury in vivo*.*^36^^–^^40^ The direct neurotrophic effect of tacrolimus is mediated by the chaperone-like FK506-binding protein 52 (FKBP52)^33^^,^^35^^,^^41^^,^^42^ forming heterocomplexes with the 90-kDa heat-shock protein 90 and its co-chaperone p2342 in the neuronal nucleus. In injured neurons, this complex redistributes to the growth cones of regenerating neurites upon cellular contact with tacrolimus, prompting their accelerated regeneration.^42^ Further, FKBP52 mediates neuronal growth cone guidance in response to attractive and repulsive chemotactic signals.^43^ Accordingly, systemically delivered tacrolimus accelerates the axonal regeneration process in vivo by 12% to 16%.^44^^,^^45^ We have previously shown that sustained local delivery of low-dose tacrolimus directly at a nerve repair site increases the number of regenerating nerve fibers and accelerates their rate of regeneration in injured peripheral nerves.^46^^–^^48^ Here, we adapted this approach to promote reinnervation of denervated corneas. We first determined the effect of local, low-dose tacrolimus therapy on the regeneration of sensory nerve fibers in a low-NGF environment in vitro and then tested the safety and efficacy of sustained topical release of tacrolimus on corneal reinnervation in vivo.

## Methods

### Study Design

The primary objective of the in vitro experiments was to determine the effect of locally delivered low-dose tacrolimus on sensory axon regeneration in a low-NGF environment. For statistical analysis, individual cultures were considered as independent samples. The experiment was repeated with true biological replicates, using cells from a different animal and fresh reagents to ensure data robustness. The number of cultures needed for these experiments was determined by power analysis based on previous experience with this system.^49^ A 50% increased axon length was considered scientifically relevant, requiring a total of nine samples per group to achieve a power of 0.8 when assuming a standard deviation (SD) of 0.3 mm for all groups (normally distributed, two-sided).

The primary objectives of the animal experiments were to determine drug biodistribution and the therapeutic efficacy of topically delivered low-dose tacrolimus on corneal reinnervation. All animal experiments are reported in accordance with the Animal Research: Reporting of In Vivo Experiments (ARRIVE) guidelines.^50^ Given the large in vitro effect sizes, a 50% increased central corneal nerve fiber density in the treatment group was considered relevant, requiring six animals each for the treatment and control groups to achieve a power of 0.8 when assuming a SD of 20% of the mean for all groups (normally distributed, two-sided). Animals were randomly allocated to experimental groups following denervation, and the investigators were blinded during outcome assessments. No experimental animals or outliers were excluded from the analysis.

### Compartmentalized Cell Cultures

Sensory neurons were cultured as previously described.^49^ Briefly, freshly harvested E15 dorsal root ganglia (DRG) were trypsinized, suspended in neurobasal (NB) media (21103049; Thermo Fisher Scientific, Waltham, MA), methylcellulose (S25427; Thermo Fisher Scientific), and 50 ng/mL NGF (11503610, Thermo Fisher Scientific). Per plate, 2500 cells were seeded into the central cell body compartment precoated with Matrigel (354234; Thermo Fisher Scientific), and poly-d-lysine (P7405; Sigma-Aldrich, St. Louis, MO). After overnight incubation, non-neuronal cells were eliminated with 10 ng/mL cytosine arabinoside (C1768; Sigma-Aldrich) for 2 days. Then, all compartments were thoroughly washed three times, 30 minutes each, and the NB/methylcellulose/NGF media added to the cell body compartment again. In cultures with axons that started to cross the barrier to the axonal compartment, the medium in the axonal compartments was changed to NB/methylcellulose containing either 50 ng/mL NGF (positive control) or 50 ng/mL tacrolimus (LC Laboratories, Woburn, MA) and 0.003% acetonitrile (1401-7-40; Caledon Laboratories, Georgetown, ON, Canada) representing the treatment group or 0.003% acetonitrile (vehicle). After 48 hours of incubation, the cultures were fixed in 4% precooled paraformaldehyde (PFA) and stained against the neuronal marker β-tubulin III using rabbit Anti-Beta III Tubulin antibody neuronal marker (ab18207, 1:500 dilution; Abcam, Cambridge, UK) with Invitrogen Goat anti-Rabbit IgG (H+L) Cross-Adsorbed Secondary Antibody, Alexa Fluor 555 (A-21428, 1:1000; Thermo Fisher Scientific). Then, cultures were imaged using an Axio Zoom.V16 microscope (Carl Zeiss Microscopy, White Plains, NY) equipped with a 2.3/0.57 objective and a pco.edge 4.2 bi XU camera (PCO Imaging, Kehlheim, Germany).

### Fabrication of the Drug Delivery System

The biodegradable polycarbonate urethane (PCNU) polymer was synthesized of butanediol (309443; Sigma-Aldrich), poly(hexamethylene carbonate) diol (461172; Sigma-Aldrich), and hexane diisocyanate (52649; Sigma-Aldrich), in a 1:2:3 ratio as previously described.^51^ For electrospinning, polymer solutions were made by mixing the PCNU polymer with 1,1,1,3,3,3-hexafluoroisopropanol (105228; Sigma-Aldrich) in concentrations of 20 w/v% for the outer shell and 14 w/v% for the inner core. Tacrolimus was added to the core polymer solution at a concentration of 5.2 w/w%. A NanoSpinner system (Inovenso Technology, Cambridge, MA) equipped with a coaxial nozzle of two concentric 18- and 22-gauge blunt-tipped needles was used to manufacture the nanofiber matrices. A constant voltage difference of 17 kV (nozzle tip to collector; 1 kV/cm) and a flow rate of 0.5 mL/h were maintained. Electrospun matrices were vacuum dried for 72 hours at room temperature, sectioned to 2.5 × 0.5-mm drug delivery systems (DDSs), wrapped in sterilization pouches, gamma irradiation sterilized (25 kGy; Southern Ontario Centre for Atmospheric Aerosol Research, University of Toronto, ON, Canada), and subsequently stored at 4°C. Liquid chromatography–tandem mass spectrometry (LC-MS/MS) was used to determine tacrolimus loading as previously reported.^52^ Drug release was determined from six DDSs derived from three different matrices. Samples were incubated in sterile phosphate buffered saline (PBS) in a water bath shaker (37°C, 50 rpm), and the solution was collected for tacrolimus content analysis via LC-MS/MS. Drug release was calculated as released tacrolimus mass per sample per day. The results of the release study have been previously reported.^52^

### Experimental Animals

A total of 24 adult (250–300 g) female rats with a genetic Sprague Dawley background were included. All animals were housed in a central animal care facility with fresh water and pellet food ad libitum. A constant room temperature (22°C) and a circadian rhythm of 12 h/24 h illumination were automatically maintained. All procedures were performed in strict accordance with the National Institutes of Health guidelines, the Canadian Council on Animal Care, and the ARVO Statement for the Use of Animals in Ophthalmic and Vision Research and were approved by the Hospital for Sick Children's Laboratory Animal Services Committee.

### In Vivo Biodegradation and Biocompatibility Testing

Biodegradation and biocompatibility were tested in eight rats. All ophthalmic and surgical procedures were performed under aseptic conditions and inhalation anesthesia with an isoflurane (Baxter, Deerfield, IL) oxygen mixture and analgesia (4 mg/kg body weight extended-release Metacam; Boehringer Ingelheim, Ingelheim, Germany). A DDS was divided into two equally sized portions (1.25 × 0.5 mm), which were applied to the superior and inferior conjunctival fornices of the left eye. A subsequent tarsorrhaphy (5-0 Prolene; Ethicon, Raritan, NJ) was performed as previously described,^13^ and animals were allowed to recover in a warm environment prior to returning to the housing facility. After 7 and 21 days, the tarsorrhaphies in four randomly selected animals were opened and the DDSs and mucous rheum were collected for light microscopic analysis using the Axio Zoom.V16 microscope. The corneas were immediately digitally imaged (D5100 DSLR Camera; Nikon, Tokyo, Japan) and analyzed for adverse local reactions. Then corneas were stained with fluorescein^13^ (Diofluor Strips; Innova Medical Ophthalmics, North York, ON, Canada) and digitally imaged with the Nikon D5100 to analyze epithelial integrity.

### Therapeutic Efficacy Testing in a Rat Model of Neurotrophic Keratopathy

A total of 16 rats underwent unilateral corneal denervation as previously described.^13^ Briefly, rats were mounted on a stereotactic frame (Harvard Apparatus, Hollingston, MA), and a midline cranial incision was made to identify bregma, representing the intersection of the coronal and sagittal sutures. A 1-mm burr hole was made at 1.5 mm anterior–posterior and 2.0 mm mediolateral, and an insulated 22-gauge monopolar electrode (UP 3/50; Pajunk GmbH, Geisingen, Germany) with 1 mm of insulation removed from the tip was lowered through the burr hole. Then, stereotactic electrocautery of the ophthalmomaxillary division of the trigeminal nerve was performed using an electrosurgical generator (Force FX-8C; Medtronic, Minneapolis, MN) with 10 W for 60 seconds. The electrode was removed and the skin sutured (6-0 Vicryl; Ethicon). Corneal denervation was confirmed by an absent blink reflex under light anesthesia after confirming an intact reflex on the contralateral eye. Then rats were block randomized to either the treatment or control group (*n* = 8 each). The treatment group received the tacrolimus DDSs as described earlier, followed by a tarsorrhaphy. The control group received only the tarsorrhaphy. After 21 days, the eyes were reopened and carefully irrigated with sterile saline, and the treatment group received fresh DDSs before the tarsorrhaphy was reclosed in all rats (control and treatment). Four weeks after corneal denervation, the rats were sacrificed, and the corneas were harvested for analysis of nerve fiber density. The 4-week time frame is based on previous experiments with this rat model of corneal denervation, indicating that this time is required for axons to reach the center of the cornea after stereotactic trigeminal nerve ablation. Briefly, the corneas were fixed for 2 hours at 4°C in precooled Zamboni's fixative consisting of 2% PFA/15% picric acid (197378; Sigma-Aldrich) in 1× PBS. Then, samples were permeabilized in 4% (w/v) sodium dodecyl sulfate (L3771; Sigma-Aldrich) in 200-mM borate (B6768, boric acid in milli-Q H_2_O, pH adjusted to 7.0; Sigma-Aldrich)^53^ at 36°C overnight, washed in 1× PBS, and immunostained against the neuronal marker β-tubulin III with a nuclear counterstaining. The following antibodies were used: rabbit Anti-Beta III Tubulin antibody neuronal marker (1:300 dilution) with Invitrogen Goat anti-Rabbit IgG (H+L) Cross-Adsorbed Secondary Antibody, Alexa Fluor 555 (1:500) and 4′,6-diamidino-2-phenylindole, dihydrochloride (DAPI, D1306, 1:500; Thermo Fisher Scientific). Corneas were imaged with a 1-µm *z*-step interval using a Leica SP8 LIGHTNING Confocal Microscope (DMI8; Leica Microsystems, Wetzlar, Germany) equipped with Leica LAS software, a Leica HyD hybrid detector, a PCO Imaging pco.edge 5.5 camera, and a 20×/0.75 (W) objective. Two regions of interest were defined for analysis in each cornea: a 1550 × 1550-µm region in the geometric center of the cornea and a randomly selected limbal region (1800 × 300 µm) capturing the entire thickness of the cornea. For data processing, the Leica Application Suite was used for three-dimensional tile stitching and Imaris 9.5.1 was used for image segmentation and quantitative morphometric analyses. Corneal nerve fibers were semiautomatically traced using batch processing with consistent fluorescence intensity thresholds across the entire dataset. Volumetric nerve fiber density was determined as total nerve fiber volume per corneal tissue volume for each region of interest. Nerve fiber length was determined as total length of the traced filaments per region of interest. The subbasal/epithelial layer was delineated from the stroma at the deepest layer of DAPI-stained epithelial cell nuclei. Selected images in this publication were created with BioRender.com.

### Tacrolimus Biodistribution Analysis

Tacrolimus biodistribution following sustained topical delivery was tested in four randomly selected rats from the therapeutic efficacy study. Four weeks after denervation, and 1 week after DDS renewal, the animals were sacrificed, and the following organs were harvested for analysis and snap frozen in liquid nitrogen: blood plasma, kidneys, heart, liver, brain, vitreal body, lens (ipsi- and contralateral), and trigeminal ganglia (ipsi- and contralateral). Prior to analysis, tissues were weighed, immersed in 300 µL lysis buffer containing 50-mM Tris (17926; Thermo Fisher Scientific), 150-mM NaCl, 2-mM EDTA (E9884; Sigma-Aldrich), 0.1% sodium dodecyl sulfate (L3771; Sigma-Aldrich), 1% NP-40 (Sigma-Aldrich), and 1% protease inhibitor, and homogenized by sonication on ice for 30 seconds twice.^47^ Tacrolimus was extracted with acetonitrile. The solution was homogenized for 30 seconds on ice twice and centrifuged at 4°C for 5 minutes at 16,000*g*, and the supernatant was then extracted for analysis with LC-MS/MS.^47^ To account for tissue matrix effects for each tissue type, we analyzed tissue from littermates (*n* = 3) of the experimental rats that were not exposed to the drug in vivo and added a known amount (2 µg) of tacrolimus to the sample before homogenization and analysis. Reported tacrolimus concentrations were adjusted accordingly.

### Statistical Analysis

We used Prism 9 (GraphPad Software, San Diego, CA) for statistical analysis. Descriptive statistics were calculated, and means are expressed with standard deviations. To test for normality of continuous variables, we used normal quantile plots and Shapiro–Wilk tests. For between-group comparisons, one-way analysis of variance (ANOVA) was conducted with Tukey's multiple comparison tests. A significance level of 5% was used (*P* < 0.05).

## Results

### Local Delivery of Tacrolimus Promotes Axonal Growth In Vitro

**Figure 1.**
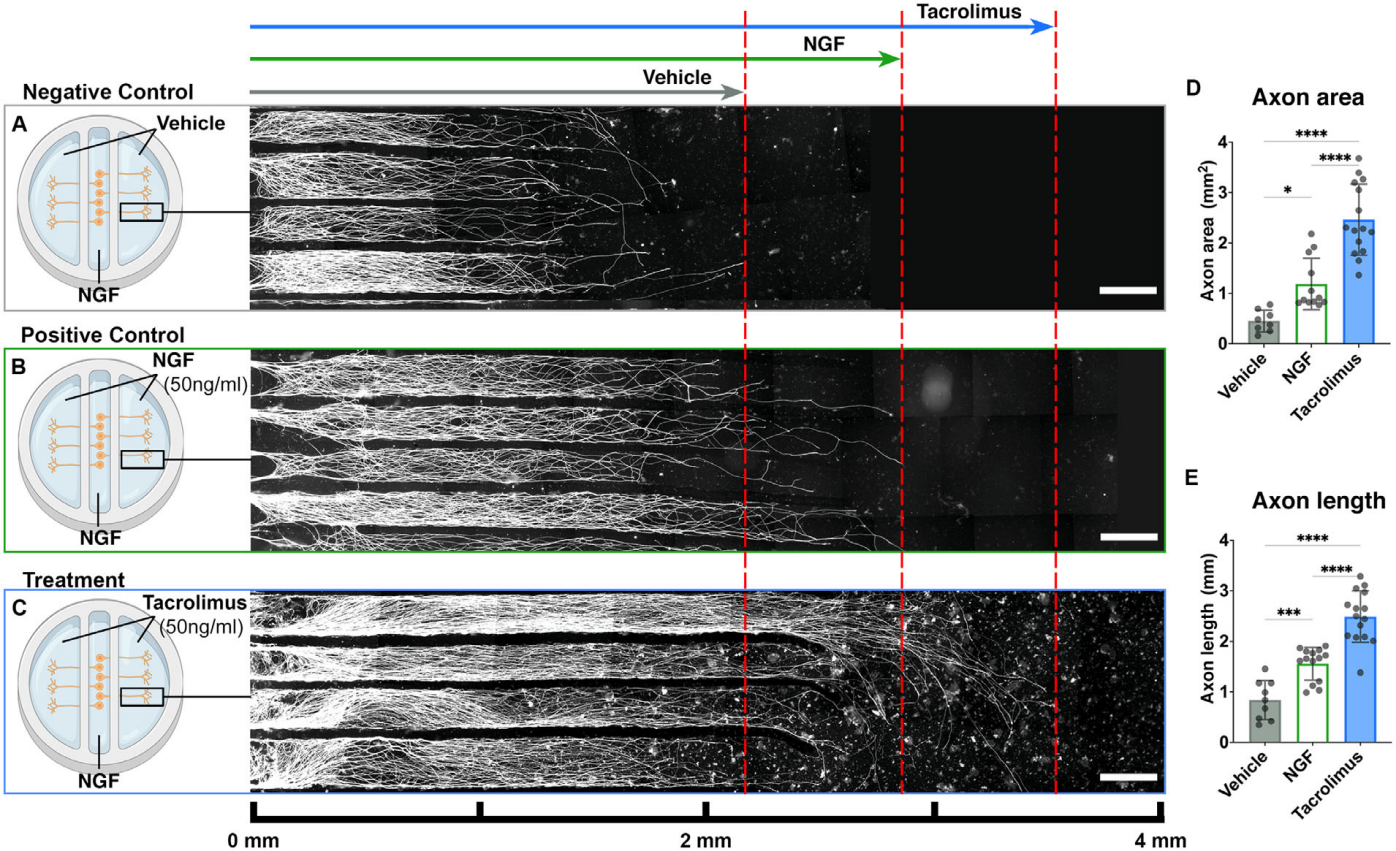
Local delivery of tacrolimus promotes sensory axon growth in vitro. E15 DRG sensory neurons were seeded in the central cell body compartment of each culture and allowed to regenerate their axons in the presence of 50 ng/mL NGF initially. Axon elongation was directed toward the side compartments via vertical barriers. The axonal compartments on both sides were physically separated from the central cell body compartment by fluid-impermeable barriers which allowed the outgrowing axons to penetrate. In all groups, a constant 50-ng/mL NGF level was maintained in the cell body compartment to ensure neuronal survival. In the axonal compartments, NGF was thoroughly washed out after axons had started to cross the barrier. (A) In negative control cultures, the axonal compartments were filled with media containing a vehicle leading to sparsely distributed, slowly elongating axons (immunostainings against the neuronal marker β-tubulin III are shown in *white*). *Scale bars*: 500 µm. (B) In the positive control group, a 50-ng/mL NGF level was maintained in all compartments, resulting in significantly longer axons with a larger surface area compared to the vehicle cultures (*P* < 0.05). (C) In the treatment groups, the axons were exposed to 50 ng/mL tacrolimus for 48 hours, leading to significantly longer axons with a significantly larger surface area compared to both control groups (*P* < 0.001). This indicates that locally delivered tacrolimus exerts a potent growth-promoting effect in regenerating axons in a low-NGF environment. (D) The total axon surface area in the axonal compartments and (E) the mean length of the axons, representing the regeneration distance within 48 hours of exposure for the three groups: vehicle, NGF, and tacrolimus.

In contrast to systemic delivery, topical application of tacrolimus to the ocular surface selectively exposes the terminal nerve fibers but not the distant neuronal cell bodies. We simulated topical tacrolimus delivery in vitro in a well-established compartmentalized primary DRG neuron cell culture system^49^^,^^54^ that allowed for isolated tacrolimus delivery (50 ng/mL) to the axonal compartment without cell body exposure (Figs. 1A–1C). A low-NGF environment for the axons was created by thorough washing as soon as the axons had crossed the border to the side compartments. The axonal compartments that were exposed to NGF (50 ng/mL) or a vehicle served as positive and negative controls, respectively (Figs. 1A–1C). After 48 hours of drug exposure, the tacrolimus-treated compartments showed significantly longer axons with a significantly greater axonal surface area compared to both positive and negative control compartments (Figs. 1D, 1E). This observation indicates that local delivery of tacrolimus promotes the axonal growth of sensory axons. Based on these results, we hypothesized that the sustained, topical application of tacrolimus to the ocular surface may exert a regenerative, growth-promoting effect on the corneal nerve fiber population and thus may improve insufficient corneal innervation.

### A Biodegradable Nanofiber Matrix Enables Sustained Topical Release of Tacrolimus

**Figure 2.**
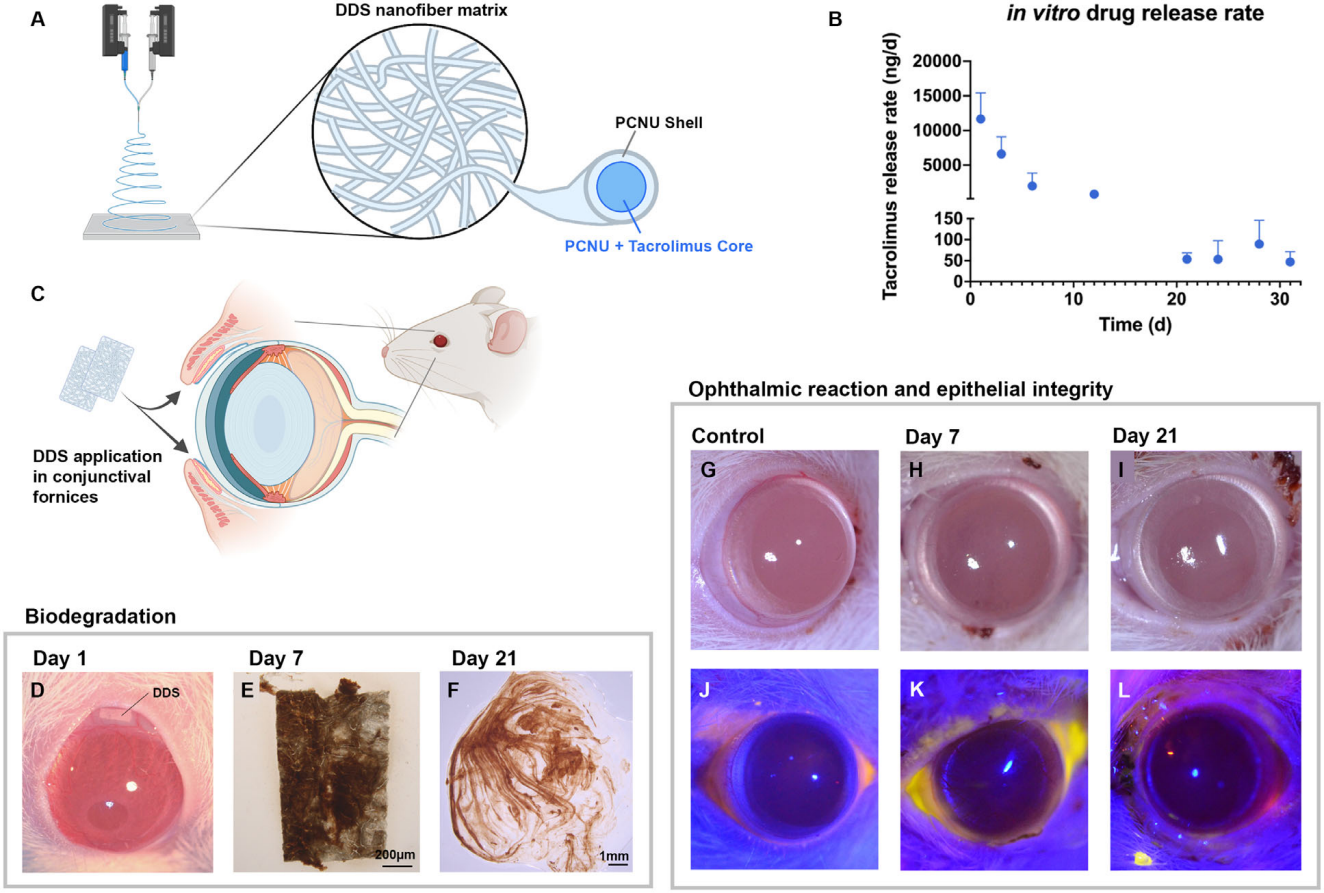
Tacrolimus drug delivery system. (A) A biodegradable PCNU polymer was electrospun into a matrix of core–shell nanofibers. Tacrolimus (5.2 w/w%) was encapsulated in the core of each fiber to create a drug reservoir for sustained release. The electrospun matrices were vacuum dried, sectioned into DDSs (2.5 × 0.5 mm), and irradiation sterilized (25 Gy) prior to application. (B) In vitro tacrolimus release profile of the DDS. An initial peak release of up to 11.7 ± 3.8 µg/d was aimed at rapidly creating a therapeutic drug concentration locally, with a subsequent maintenance dose averaging 60.7 ± 37.3 ng/d on days 12 to 31. These data and a detailed characterization of the DDS have been published previously.51 (C) Application of the DDSs (2 × 100 µg tacrolimus) into the upper and lower conjunctival fornix, with a subsequent protective tarsorrhaphy. (D) DDS placed in the upper conjunctival fornix of an adult rat. Note the initial conjunctival injections in response to manipulation during DDS placement which were absent when reopening the tarsorrhaphy after 7 and 21 days. (E) Partially degraded DDS after 7 days of ophthalmic application. (F) After 21 days of application, the DDS was entirely absorbed in all animals (*n* = 4), leaving behind a mucous rheum. (G–I) Digital macroscopic imaging of a healthy control cornea (G), a cornea after 7 days of DDS application (H), and a cornea after 21 days of DDS application (I), showing no signs of adverse local reaction to DDS application. (J–L) Corneal fluorescein staining of a healthy control cornea (J), a cornea after 7 days of DDS application (K), and a cornea after 21 days of DDS application (L), showing an intact corneal surface without epithelial defects that appear in *yellow*.

To deliver tacrolimus locally we aimed at developing a biocompatible and biodegradable DDS suitable for ophthalmic application yet minimizing systemic drug exposure and systemic side effects of tacrolimus. Based on our previous work, we used electrospinning to manufacture a core–shell nanofiber matrix from a biocompatible and biodegradable polycarbonate–urethane polymer (Fig. 2A).^52^ Tacrolimus was encapsulated in the core of each fiber to create a sustained-release profile. Detailed physical characterization, drug release kinetics, and bioactivity studies for this biodegradable nanofiber DDS have been reported previously.^52^ Briefly, the electrospun matrices were sectioned in 0.5 × 2.5-mm sheets, yielding 223 ± 65 µg tacrolimus each. The DDSs were subsequently irradiation sterilized (25 Gy) and stored at 4°C until use. In vitro drug release studies confirmed tacrolimus delivery for at least 31 days, with an initial peak release rate of 11.7 ± 3.8 µg/d and a subsequent maintenance dose averaging 60.7 ± 37.3 ng/d on days 12 to 31 (Fig. 2B). Biocompatibility and biodegradation for corneal application were assessed by dividing each DDS into two equally sized microsheets (0.5 × 1.25 mm) and placing them into the superior and inferior conjunctival fornix of rats, respectively, with a subsequent tarsorrhaphy (Figs. 2C, 2D). Compared to the DDS degradation after implantation around peripheral nerves, the degradation was considerably faster when applied to the ocular surface. After 7 days, the DDSs showed significant signs of degradation, including thinning and partial disintegration (Fig. 2E); after 21 days, the DDSs were entirely degraded in all animals (Fig. 2F). Further, local adverse effects of the DDS application, such as inflammation, conjunctival injection (Figs. 2G–2I), or epithelial defects (Figs. 2J–2L), were not observed.

### Topically Delivered Tacrolimus Permeates the Ocular Surface With Minimal Systemic Exposure

The cornea represents a dense physical barrier,^55^ with an enzyme-rich tear film with a high turnover rate,^56^ which may decompose drug molecules rapidly before they can penetrate the ocular surface and exert a therapeutic effect. To determine the biodistribution of tacrolimus following sustained, topical delivery, we used liquid chromatography–tandem mass spectrometry. Aiming at a 4-week duration of treatment for promoting corneal nerve fiber regrowth, we reopened the tarsorrhaphy after 3 weeks, irrigated and inspected the eye, and applied new tacrolimus DDSs in a manner similar to that of the initial procedure.

**Figure 3.**
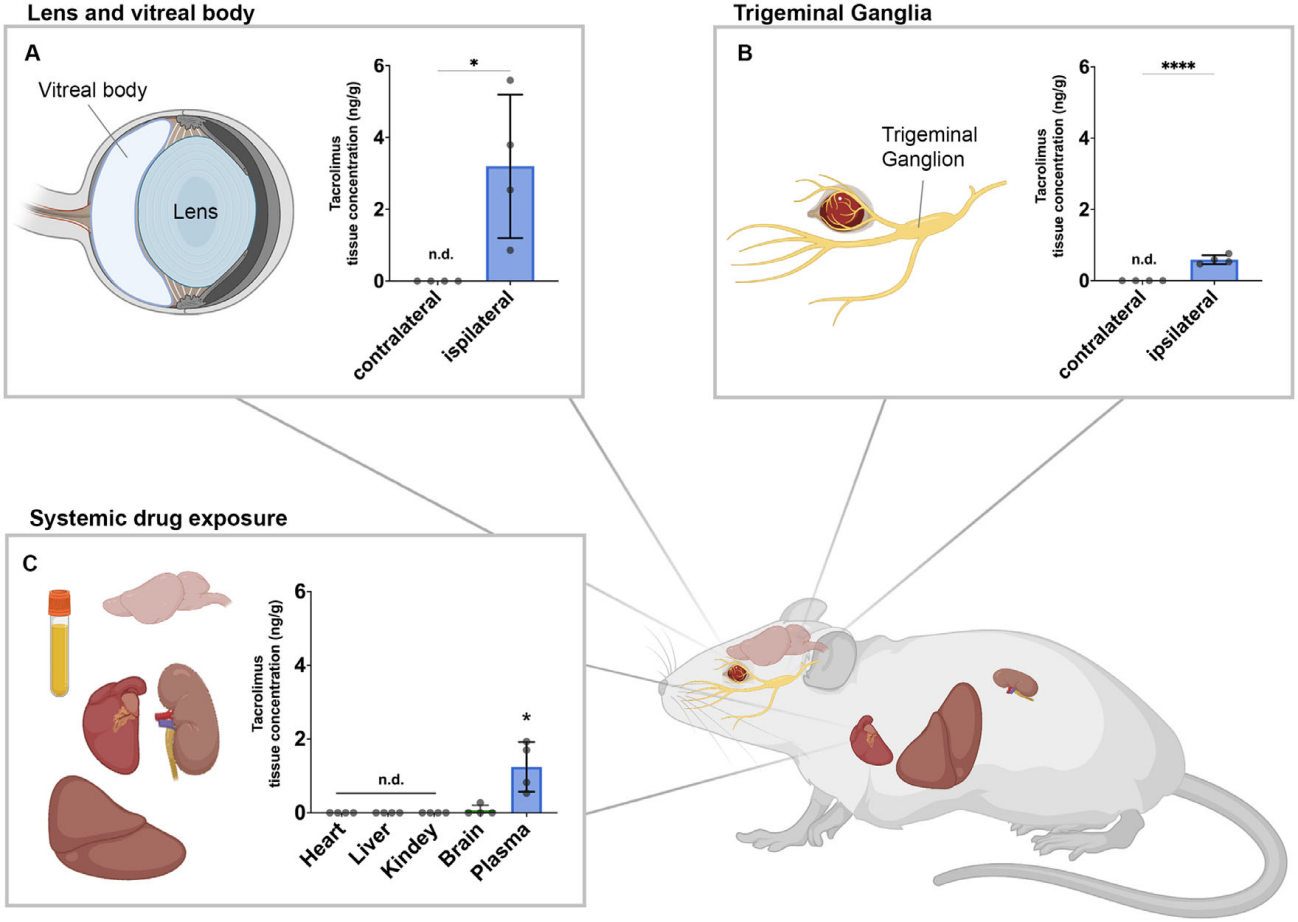
Tacrolimus biodistribution following sustained topical release. (A) After 4 weeks of topical tacrolimus release (total dose, 400 µg), the lens and vitreal body of both eyes were harvested and analyzed for their tacrolimus tissue concentration via LC-MS/MS. Tacrolimus was present in the ipsilateral but not contralateral eye, indicating tacrolimus penetration through the ocular surface and thereby likely exposing the corneal nerve fibers to the drug. (B) Sensory neurons that project their axons into the cornea are located in the ipsilateral trigeminal ganglion.^61^ Tacrolimus was found in the ipsilateral but not the contralateral trigeminal ganglion, which may suggest a retrograde transport of the drug. (C) Tacrolimus was present in the blood plasma, and traces were detectable in the brain; however, in vital organs known to be susceptible to side effects of tacrolimus including heart, liver, and kidney, tacrolimus was not detectable following 4 weeks of topical application. This agrees with previous studies indicating that topical ophthalmic application of tacrolimus is safe.

Four weeks after the initial DDS application, the ipsi- and contralateral lens and vitreal bodies were analyzed for their tacrolimus tissue concentration (Fig. 3A). Significant amounts of tacrolimus were detected in the ipsilateral but not the contralateral eye, indicating drug penetration through the ocular surface. From previous experiments with regenerating peripheral nerves, we learned that locally delivered tacrolimus accumulates in the neuronal cell bodies, suggesting a retrograde transport of the drug.^47^ Similarly, tacrolimus was detectable in the ipsilateral but not the contralateral trigeminal ganglion after topical ophthalmic application, demonstrating that corneal nerve fibers incorporate and retrogradely transport tacrolimus to their neuronal cell bodies.

Systemic exposure to tacrolimus can cause side effects including nephro- and hepatotoxicity,^57^^,^^60^ as well as neurologic^59^ and psychiatric^60^ symptoms. We therefore determined the systemic exposure following a sustained 4-week topical tacrolimus delivery by conducting a drug content analysis of blood plasma and vital organs known to be susceptible to tacrolimus. Tacrolimus was detected in the blood plasma, and traces were also detected in the brain of rats treated with topical tacrolimus (Figs. 3B, 3C).^61^ However, tacrolimus was undetectable in the heart, liver, and kidney, indicating minimal systemic exposure through sustained topical drug release.

### Sustained, Topical Delivery of Tacrolimus Enhances Corneal Reinnervation

**Figure 4.**
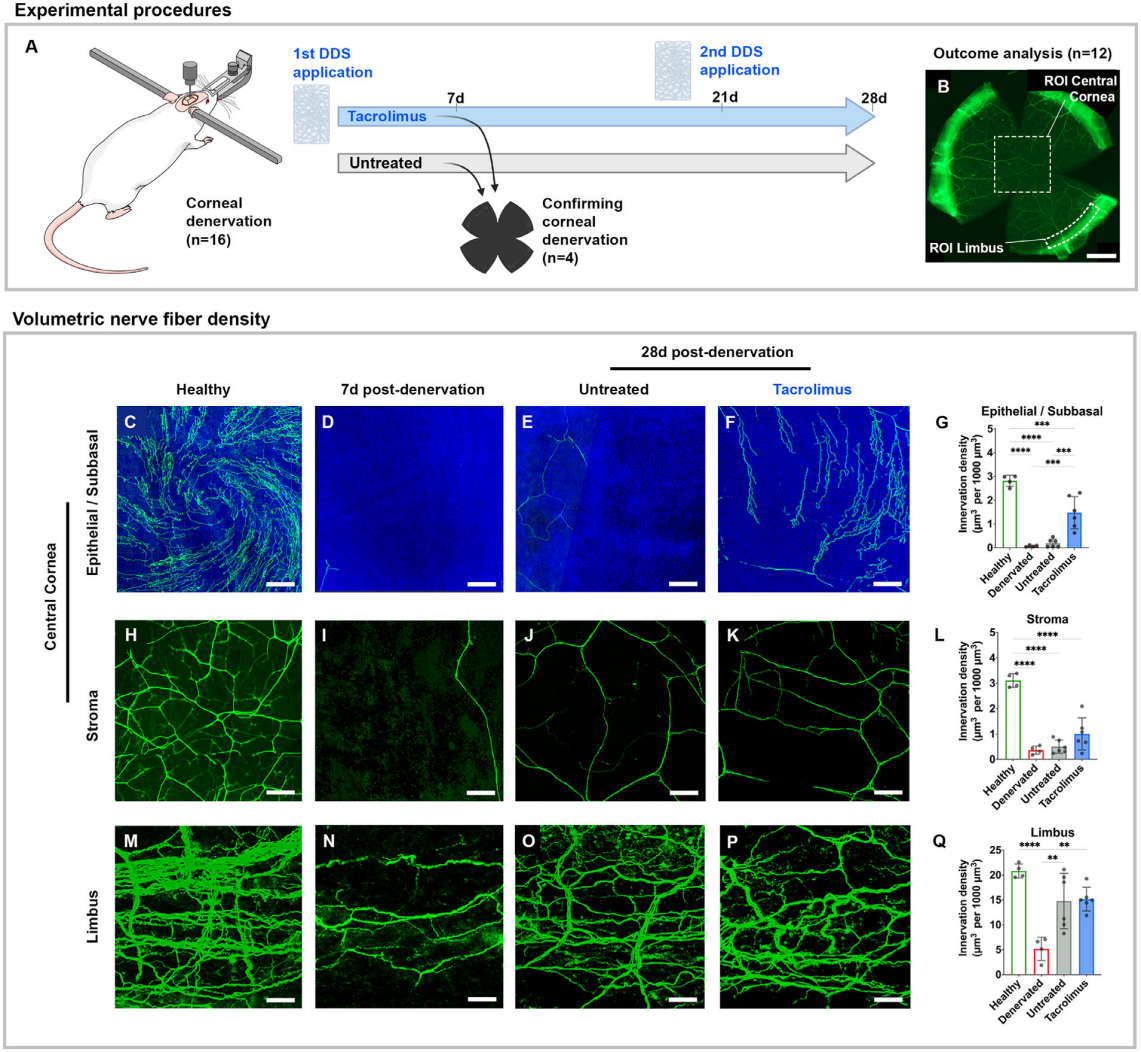
Sustained topical release of tacrolimus promotes corneal reinnervation. (A) Adult rats underwent unilateral ophthalmic nerve ablation to induce corneal denervation and were subsequently block randomized into treatment and control groups (*n* = 8 each), receiving either the tacrolimus-releasing DDS immediately before tarsorrhaphy or a tarsorrhaphy only. Corneal denervation was confirmed 7 days later in two randomly chosen rats of each group. After 21 days, the DDSs were renewed in the treatment group. Corneal reinnervation was analyzed 28 days after denervation. (B) A control cornea 28 days after denervation immunostained against the neuronal marker β-tubulin III (*green*). For the analysis of the volumetric corneal nerve fiber density, regions of interest (ROIs) were defined in the geometric center of the cornea (epithelial/subbasal layer, 1550 × 1550 × 20 µm; stromal layer, 1550 × 1550 × 50 µm) and a representative section of the limbus (1800 × 300 × 70 µm). *Scale bar*: 750 µm. (C–F) Epithelial/subbasal innervation in the center of a normal cornea (healthy), a cornea 7 days after denervation (denervated), and a cornea 28 days after denervation, either with no treatment (untreated) or after 4 weeks of topical tacrolimus treatment (tacrolimus). *Scale bar*: 50 µm. DAPI, *blue*; β-tubulin III, *green*. (G) Volumetric innervation density of the subbasal/epithelial plexus in the center of the cornea showing significantly higher nerve fiber density in tacrolimus-treated eyes (*P* < 0.001). (H–K) Stromal innervation in the center of the cornea. (L) Volumetric innervation density of the stromal plexus in the center of the cornea. The difference between treated and untreated corneas did not reach statistical significance (*P* = 0.18). (M–P) Limbal innervation of normal cornea (healthy) 7 days after denervation (denervated) and 28 days after denervation, either with no treatment (untreated) or after 4 weeks of topical tacrolimus release (tacrolimus). (Q) Volumetric innervation density of the limbal plexus indicating rapid recovery of limbal innervation within 4 weeks after denervation in treated and untreated rats. Images D, I, and N are representative for treated and untreated rats.

**Figure 5.**
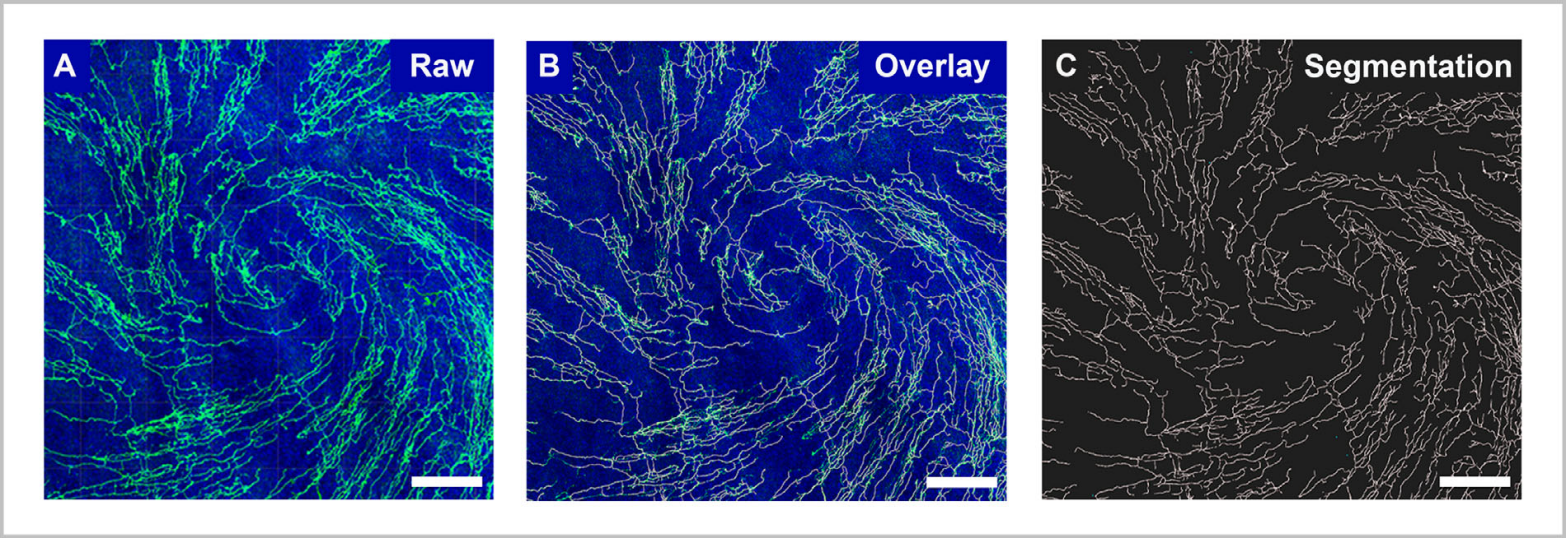
Volumetric image segmentation. A standardized pipeline for automated volumetric image segmentation based on the fluorescence intensity of β-tubulin III immunolabeled nerve fibers allowed for comparative histomorphometry of corneal innervation. (A) A representative raw image of the epithelial/subbasal layer in the center of a healthy cornea. (B) The result of the automated nerve fiber segmentation as a *gray overlay*. (C) Segmentation only. *Scale bar*: 50 µm. DAPI, *blue*; β-tubulin III, *green*; segmented nerve fibers, *gray*.

To determine the effect of a 4-week, sustained, topical delivery of tacrolimus, we used a rat model of NK.^13^ The rats underwent stereotactic ablation of the ophthalmic nerve to induce complete corneal denervation, which was intraoperatively confirmed by loss of corneal sensation compared to the contralateral side. The rats were then block randomized to two experimental groups. A treatment group received two DDSs loaded with 100 µg tacrolimus each, which were each placed into the superior and inferior conjunctival fornix. Untreated animals served as a control. To protect the insensate cornea from injury, all rats received a tarsorrhaphy immediately after denervation. One week after surgery, two rats were randomly selected from each group to confirm corneal denervation and quantify the density of the remaining corneal nerve fibers. After 3 weeks, the tarsorrhaphy was reopened in all experimental rats, each eye was gently irrigated, and the degraded DDSs in the treatment group were replaced with fresh DDSs, followed by a protective tarsorrhaphy for all animals (experimental procedures are summarized in Fig. 4^5^A). Four weeks after denervation, the corneas were harvested (Fig. 4B) and assessed for reinnervation via β-tubulin III immunohistochemistry, confocal microscopy, and digital image segmentation (Fig. 5).

Corneal nerves usually emerge from the limbal plexus in bundles within the stromal layer and run centripetally toward the center of the cornea, where the majority end as terminal nerve fibers in the superficial epithelial layers (Figs. 4A, 4H, 4M).^62^^,^^63^ One week after denervation, the nerve fiber densities in the limbus and geometric center of the cornea were reduced to 24.9% and 9.3% of the normal corneal innervation density, respectively (*P* < 0.0001) (Figs. 4D, 4I, 4N). The greatest nerve fiber loss was observed in the superficial layers of the central cornea, with the nerve fiber density of the subbasal and epithelial plexus dropping to 2.6% of their pre-denervation state (Figs. 4D, 4G). Four weeks after denervation, regenerating nerve fibers had reinnervated the cornea in both the untreated control group and the treatment group that had received the tacrolimus DDSs. In the control and tacrolimus-treated rats, the limbal nerve fiber plexus recovered to 71.0% and 72.8% of its normal innervation density, respectively (*P* = 0.86) (Figs. 4O–4Q). In contrast, the tacrolimus-treated rats showed a significantly higher density of nerve fibers in the center of the cornea compared to untreated rats (37.6% vs. 13.7% of the normal innervation density; *P* = 0.0009). The difference was most evident in the subbasal/epithelial layers of the corneal center, where corneal axons terminate and therefore had to regenerate the farthest (Figs. 4E–4G). Similarly, subbasal axons in animals that had received the tacrolimus-releasing DDSs were significantly longer compared to non-treated animals 4 weeks after denervation (*P* = 0.0043), suggesting accelerated corneal reinnervation.

## Discussion

Here, we have demonstrated that the local delivery of low-dose tacrolimus to regenerating sensory axons enhances their regrowth in vitro and promotes corneal reinnervation in a rat model of surgically induced corneal denervation. Further, we have presented a biodegradable nanofiber DDS that enables the sustained release of tacrolimus to the ocular surface with minimal systemic drug exposure and demonstrated its applicability and therapeutic efficacy in vivo.

Based on recent work of our laboratory demonstrating the effectiveness of locally delivered low-dose tacrolimus to injured nerves,47 we hypothesized that topically applied tacrolimus may exert a similar growth-promoting effect on corneal nerve fibers. To address this hypothesis, we first used a compartmentalized cell culture system for sensory neurons that allowed for isolated treatment of the axonal compartment without exposing the neuronal cell bodies to the drug. We simulated a low NGF environment in vitro because poorly innervated corneas are deficient in this factor, which, in turn, impairs epithelial renewal and healing.^25^^,^^64^^,^^65^ We washed the axonal compartments thoroughly before tacrolimus or vehicle application. For the cell bodies, however, we maintained a NGF level of 50 ng/mL to ensure neuronal survival. Axons that were exposed to tacrolimus in the low-NGF environment were significantly longer (+296%) and occupied a significantly larger surface area (+547%) compared to control axons exposed to vehicle only (Figs. 1D, 1E). These data align with previously reported observations indicating that tacrolimus may increase the sensitivity of regenerating neurons to NGF and thereby elicits its potent growth promotive effect, particularly in the presence of low NGF levels.32 Further, the observed growth-promoting effect was comparable to previously reported in vitro observations where tacrolimus was delivered to both the neuronal cell bodies and axons.^32^^,^^66^ This suggests that the sensory axons that represent a large proportion of the corneal nerve fibers^2^ are receptive to local treatment with tacrolimus. Based on these results, we developed a biodegradable, local DDS that enables the sustained release of tacrolimus to the ocular surface to determine whether the drug promotes the regrowth of nerve fibers in the denervated cornea in vivo.

Based on recent work in our laboratory, we used an electrospun, core–shell nanofiber matrix composed of a biodegradable polycarbonate–urethane polymer.^52^ Tacrolimus was embedded in the core of each nanofiber to achieve a sustained-release characteristic of at least 31 days (Fig. 2B). We have previously applied this matrix as a tacrolimus-releasing implant and demonstrated its biocompatibility, reliable drug delivery*,* and full degradation into nontoxic byproducts within a clinically reasonable time.^52^ For translation to ophthalmic use, we created a 50% smaller DDS loaded with 100 µg tacrolimus and first tested its biocompatibility and biodegradability following topical application and subsequent tarsorrhaphy in a rat model. To achieve a homogeneous drug distribution, we placed two DDSs into each eye, one into the upper and one in the lower conjunctival fornix. A subsequent tarsorrhaphy protected the susceptible corneal epithelium from injury and held the DDSs in place. After 7 days, the DDSs showed significant signs of degradation, including thinning and partial disintegration (Fig. 2E). Three weeks after application, the DDSs were entirely degraded, leaving behind only a mucous rheum (Fig. 2F). This indicated a considerably faster biodegradation of the nanofiber matrix following ophthalmic application as compared to following implantation around injured peripheral nerves, where it degrades within 120 days. The accelerated biodegradation may be a result of increased mechanical strain induced by eye movements and/or the enzyme-rich, high-turnover tear film,^56^ leading to accelerated structural disintegration of the polymer. As a lipophilic drug, however, topically delivered tacrolimus may benefit from the significantly slower turnover rate of the lipid layers of the tear film, potentially contributing to prolonged corneal drug exposure.^56^ Further, we determined the local ophthalmic reaction to the DDS application. After initial conjunctival injection due to ocular manipulation during the DDS placement (Fig. 2D), we observed neither signs of adverse ophthalmic reaction nor epithelial lesions after 7 and 21 days of application (Figs. 2K, 2L). Of note, biocompatibility experiments were performed in rats with normal (healthy) corneal innervation because the frequently observed ocular automutilation following corneal denervation would likely have affected interpretation of any ocular irritation.

In the context of nerve fiber regeneration, most studies investigated systemically delivered tacrolimus.^36^^–^^39^^,^^46^^,^^67^^,^^68^ To achieve therapeutic concentrations in the non-vascularized cornea, however, systemically administered drugs require unacceptably high dosing.^69^ High doses of tacrolimus cause significant side effects, including nephro- and hepatotoxicity^57^^,^^58^ and sometimes neurologic^59^ or psychiatric^60^ symptoms, that would outweigh its anticipated benefits. Topical delivery, however, could minimize the systemic exposure while enabling a localized therapeutic drug level. To determine the biodistribution of topically delivered tacrolimus, we analyzed the tacrolimus tissue concentration after 4 weeks of topical release. Low levels of tacrolimus were detectable in the blood plasma, corresponding to a 90% reduction compared to the average 24-hour plasma concentration following systemic tacrolimus administration (2 mg/kg/d) in the same rat strain.^52^ Further, the detected tacrolimus plasma level of 1.25 ± 0.7 ng/mL was considerably lower compared to the trough concentration (C_min_) of around 10 ng/mL typically used for immunosuppression.^70^

Together, these data suggest non-toxicity of the tacrolimus levels present following ophthalmic application.^71^ Further, tacrolimus was not detectable in the heart, liver, or kidney following 4 weeks of topical delivery. In contrast to the low systemic exposure, tacrolimus accumulated in the ipsilateral but not contralateral lens and vitreal body, indicating drug penetration through the ocular surface and thereby exposure of corneal nerve fibers to tacrolimus. Moreover, we analyzed accumulation in the cell bodies of trigeminal neurons that project their axons to the cornea. Tacrolimus was detectable in the ipsilateral but not the contralateral trigeminal ganglion, suggesting a retrograde axonal transport of the drug, as previously hypothesized after local delivery to injured nerves.^47^

Based on these results, we asked whether topical sustained release of tacrolimus in a rat model of neurosurgically induced NK could improve corneal innervation.^13^ Stereotactic trigeminal nerve ablation reduced the nerve fiber density in the limbus and the geometric center of the cornea by 75% and 91% compared to the normal corneal innervation density, respectively. In preliminary experiments, we found that in this rat model a subset of the injured trigeminal nerve fibers can regenerate and reinnervate the cornea in a centripetal manner, entering the limbus and subsequently elongating toward the center of the cornea. This was reflected in the results of the corneal nerve fiber density analysis that was carried out 4 weeks following denervation. The limbal innervation density recovered to approximately 70% of the normal innervation, with no differences between tacrolimus-treated and non-treated animals (Figs. 4M–4Q). Because regenerating trigeminal nerve fibers reach the limbal aspect of the cornea first, the undetectable treatment effect on limbal nerve fiber density may be related to the timing of the outcome analysis. One could speculate that, despite a possibly earlier corneal innervation by the regenerating axons in the treatment group, the limbal innervation density in the control group might level up over time. Further, reinnervating axons may have to reach the eye first to be exposed to therapeutic levels of topically delivered tacrolimus; thus, a growth-promoting effect may be more pronounced in the central region of the cornea. Accordingly, we found that the innervation density and the total nerve fiber length in the central aspects of the cornea, and specifically in the epithelial layer, were significantly increased in tacrolimus-treated eyes compared to untreated eyes. The observed potent growth-promoting effect on epithelial nerve fibers could also be the result of higher drug levels in the corneal epithelium. Although corneal permeability in patients with NK may be increased due to impaired surface integrity,^72^ topically delivered tacrolimus likely penetrates the superficial epithelial layers more easily due to its lipophilic characteristics, as compared to the deeper, hydrophilic stromal layers.^55^ Taken together, this indicates that the sustained, topical delivery of tacrolimus promotes corneal reinnervation following trigeminal nerve lesions in rats.

A limitation of the rat model of acute corneal denervation is the rapid and spontaneous recovery of corneal innervation. This does not necessarily reflect the clinical situation in patients with NK who would potentially benefit from topical tacrolimus treatment. Therefore, future studies may investigate whether these results are translatable to chronic progressive nerve fiber loss, as in diabetic animal models and in long-term denervated corneas. Further, this preclinical study and most previously conducted preclinical in vivo experiments were conducted in rats, potentially limiting the translatability to other species. However, clinical case reports^73^^–^^75^ suggest that the growth-promoting effect of tacrolimus on regenerating nerve fibers could also be present in humans. Given the well-established clinical safety profile of tacrolimus in ophthalmology^76^^–^^78^ and other indications,^58^^,^^70^^,^^72^^,^^79^^,^^80^ this approach may therefore be readily translatable into clinical trials. Further, although convenient for patients, a dedicated drug delivery system for sustained, topical delivery of tacrolimus may not be necessary for initial clinical investigations. Tacrolimus eyedrops can be self-administered throughout the day, but with a mean surface residence time of >1.5 hours that would require very frequent dosing.^81^ Due to the required tarsorrhaphy it was not possible to use this approach in the reported rat model. However, future clinical studies will determine whether this is sufficient for maintaining therapeutic levels of tacrolimus in the cornea. As a proof-of-concept study, investigators may primarily focus on surgically denervated corneas with the confirmed potential for nerve regeneration, such as following trigeminal nerve crush injury or surgical nerve repair. Alternatively, translating this approach to patients following corneal neurotization may offer the added scientific benefits of a highly standardized procedure with a well-defined number of transferred donor nerve fibers via intraoperative nerve biopsies, a standardized regeneration distance, and known entry points of the nerve fibers into the cornea which may allow for nerve fiber tracking via in vivo confocal microscopy.

## Conclusions

In conclusion, the topical and sustained release of tacrolimus to the ocular surface may provide an effective and readily translatable approach to promote nerve fiber regrowth in patients suffering from corneal denervation and neurotrophic keratopathy.
